# Over-expression of *BCAT1*, a c-Myc target gene, induces cell proliferation, migration and invasion in nasopharyngeal carcinoma

**DOI:** 10.1186/1476-4598-12-53

**Published:** 2013-06-08

**Authors:** Wen Zhou, Xiangling Feng, Caiping Ren, Xingjun Jiang, Weidong Liu, Wei Huang, Zhihong Liu, Zan Li, Liang Zeng, Lei Wang, Bin Zhu, Jia Shi, Jie Liu, Chang Zhang, Yanyu Liu, Kaitai Yao

**Affiliations:** 1 Cancer Research Institute, Xiang-Ya School of Medicine, Key Laboratory for Carcinogenesis of Chinese Ministry of Health, Key Laboratory for Carcinogenesis & Cancer Invasion of Chinese Ministry of Education, Central South University, Xiangya Road 110, 410078, Changsha, Hunan, P. R. China; 2 Department of Neurosurgery, Xiangya Hospital, Central South University, Changsha, Hunan, P. R. China; 3 Department of Pathology, Hunan Tumor Hospital, Changsha, Hunan, P. R. China; 4 Department of Head and Neck Surgery, Hunan Tumor Hospital, Changsha, Hunan, P. R. China; 5 Cancer Research Institute, Southern Medical University, Guangzhou, Guangdong, P. R. China

**Keywords:** Nasopharyngeal carcinoma, BCAT1, c-Myc, Proliferation, Migration, Invasion, Gene amplification, Gene regulation

## Abstract

**Background:**

Nasopharyngeal carcinoma (NPC) is a common malignant tumor in southern China and Southeast Asia, but its molecular mechanisms of pathogenesis are poorly understood. Our previous work has demonstrated that *BCAT1* mRNA is over expressed in NPC and knocking down its expression in 5-8F NPC cell line can potently inhibit cell cycle progression and cell proliferation. However, the mechanism of *BCAT1* up-regulation and its functional role in NPC development remain to be elucidated yet.

**Methods:**

Immunohistochemistry (IHC) method was utilized to detect the expression of BCAT1 protein in NPC at different pathological stages. The roles of gene mutation, DNA amplification and transcription factor c-Myc in regulating *BCAT1* expression were analyzed using PCR-sequencing, quantitative polymerase chain reaction (qPCR), IHC, ChIP and luciferase reporter system, respectively. The functions of *BCAT1* in colony formation, cell migration and invasion properties were evaluated by RNA interference (RNAi).

**Results:**

The positive rates of BCAT1 protein expression in normal epithelia, low-to-moderate grade atypical hyperplasia tissues, high-grade atypical hyperplasia tissues and NPC tissues were 23.6% (17/72), 75% (18/24 ), 88.9% (8/9) and 88.8% (71/80), respectively. Only one SNP site in exon1 was detected, and 42.4% (12/28) of the NPC tissues displayed the amplification of microsatellite loci in *BCAT1*. C-Myc could directly bind to the c-Myc binding site in promoter region of *BCAT1* and up-regulate its expression. The mRNA and protein of *c*-*Myc* and *BCAT1* were co-expressed in 53.6% (15/28) and 59.1% (13/22) of NPC tissues, respectively, and *BCAT1* mRNA expression was also down-regulated in c-Myc knockdown cell lines. In addition, *BCAT1* knockdown cells demonstrated reduced proliferation and decreased cell migration and invasion abilities.

**Conclusions:**

Our study indicates that gene amplification and c-Myc up-regulation are responsible for *BCAT1* overexpression in primary NPC, and overexpression of *BCAT1* induces cell proliferation, migration and invasion. The results suggest that *BCAT1* may be a novel molecular target for the diagnosis and treatment of NPC.

## Background

Nasopharyngeal carcinoma (NPC) is a squamous cell carcinoma that develops from the epithelium of the nasopharynx with a high incidence in Southeast Asia and southern China and causes a serious healthcare problem in these regions [1]. More than 95% of NPC in southern China is undifferentiated carcinoma with a high incidence of early metastasis which is the main cause of death in NPC patients. Currently, radiation therapy is the first choice for NPC treatment. Although the radiotherapy equipments and techniques have been improved tremendously, the five-year survival rate of NPC patients has not radically changed yet and remains around 50-60%. Therefore, it is of great importance to comprehensively explore the new approaches for NPC treatment.

The molecular mechanisms of nasopharyngeal carcinogenesis have not been elucidated clearly yet. Previous studies have shown that proto-oncogenes (*e*.*g*. *HRAS*, *NRAS2*[2], *cyclin D1*[3], *MDM2*[4], *EVI1*[5], *EGFR*[6]) and tumor suppressor genes (TSGs) (*e*.*g*. *p53*[7], *p16*[8], *RASSF1A*[9], *DLC*-*1*[10], *LTF*[11,12], *DLEC1*[13], *TSLC1*[14]) are aberrantly expressed in NPC. However, none of them has been confirmed as an NPC-specific oncogene or TSG. Using comparative genomic hybridization (CGH) data from 170 primary NPC cases, we have developed a tree model indicating the pathogenetic mechanisms of NPC [15]. According to the tree model, +12p11-12 may represent an early event in the carcinogenesis of NPC [15]. We further identified that *BCAT1*, *KCNJ8*, *PTX1* and *KRAS2*, four genes located at 12p11-12, were significantly up-regulated in NPC tissues compared to the normal controls [16]. *BCAT1* (branched chain aminotransferase 1 gene, also known as *ECA39*) is also significantly up-regulated in Burkitt’s lymphoma and breast cancer [17]. Therefore, we selected *BCAT1* as a target gene for further study to explore its relationship with NPC development. In our previous work, we found that *BCAT1* mRNA expression was over expressed in NPC tissues, and *BCAT1* knockdown in 5-8F NPC cell line inhibited cell cycle progression and cell proliferation.

In this report, we further investigated the expression of BCAT1 protein in tissues at various stages including normal epithelia, mild or moderate hyperplasia, severe atypical hyperplasia and NPC. We also explored how *BCAT1* is up-regulated and its functional roles in NPC proliferation, migration and invasion.

## Results

### The expression of BCAT1 protein increased significantly at early stage of NPC

**Figure 1:**
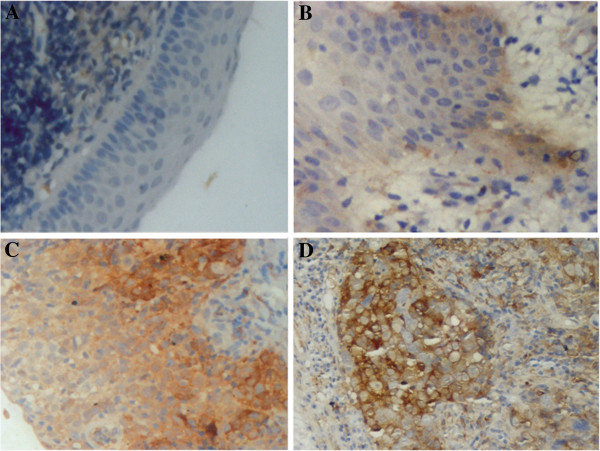
**Detection of BCAT1 protein in different pathological stages of NPC.** (**A**) Normal pseudo-stratified ciliated epithelium. (**B**) Low-to-moderate grade atypical hyperplasia tissue. (**C**) High-grade atypical hyperplasia tissue. (**D**) NPC tissue. The results demonstrated that the expression level of BCAT1 protein had increased significantly since early pathological stages of NPC.

To evaluate the significance of *BCAT1* in NPC pathogenesis, we investigated the expression of BCAT1 protein in different stages of precancerous and cancerous lesions in nasopharyngeal biopsies. Cytoplastic immunostaining signals of BCAT1 could be detected at different stages, but the positive rates differed greatly, which were 23.6% (17/72), 75.0% (18/24), 88.9% (8/9) and 88.8% (71/80) in normal epithelia, low-to-moderate grade atypical hyperplasia tissues, high-grade atypical hyperplasia tissues and NPC tissues, respectively (Figure 1, Table 1, *P* < 0.05), indicating that up-regulation of BCAT1 is an early event in NPC pathogenesis.

**Table 1:** Statistical analysis for BCAT1 expression in different stages of NPC

**Groups**	**No**.	**BCAT1**				P^*^
		**Negative**		**Positive**		
		**—**	**+**	**++**	**+++**	
		**No.(%)**	**No.(%)**	**No.(%)**	**No.(%)**	
Normal epithelia	72	42(58.3)	13(18.1)	17(23.6)	0	
Low-to-moderate grade atypical hyperplasia tissues	24	5(20.8)	1(4.2)	18(75)	0	0.000
High-grade atypical hyperplasia tissues	9	1(11.1)	0	5(55.6)	3(33.3)	0.000
NPC tissues	80	7(8.7)	2(2.5)	58(72.5)	13(16.3)	0.000

P* value was calculated by comparing the positive rate of BCAT1 in low-to-moderate grade atypical hyperplasia tissues, high-grade atypical hyperplasia tissues and NPC tissues with that in normal epithelia, respectively.

### No mutation of *BCAT1* was found in NPC tissues

**Figure 2:**
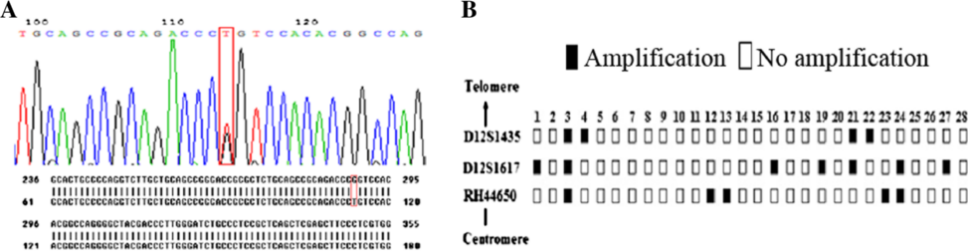
**Exon mutation and amplification of *BCAT1*.** (**A**) BLAST analysis result of *BCAT1* exon 1. The red box indicates SNP site (+78G/T) by DNA sequencing. (**B**) The amplification status of three *BCAT1* microsatellite loci in NPC samples, showing that the amplification ratios for D12S1435, D12S1617 and RH44650 were 14% (4/28), 25% (7/28) and 17% (5/28), respectively, and the total ratio was 42.4% (12/28).

Since gene mutation and DNA amplification are two major causes for oncogene up-regulation, we first performed DNA sequencing of the full-length of 11 exons in *BCAT1*. Only one polymorphism (G/T) was detected at +78 in the non-coding region of first exon (Figure 2A), which was further confirmed in the single nucleotide polymorphism (SNP) database.

### Frequent amplification of *BCAT1* was detected in NPC tissues

Three microsatellites (D12S1435, D12S1617 and RH44650) located within *BCAT1* gene were selected for analysis of *BCAT1* amplification. Real-time PCR was employed to detect DNA samples from 28 NPC tissues and their matched peripheral blood specimens. The amplification ratios of D12S1435, D12S1617 and RH44650 were 14% (4/28), 25% (7/28) and 17% (5/28), respectively (Figure 2B). The total amplification ratio was 42.4% (12/28).

### The transcription factor c-Myc regulated *BCAT1* expression

**Figure 3:**
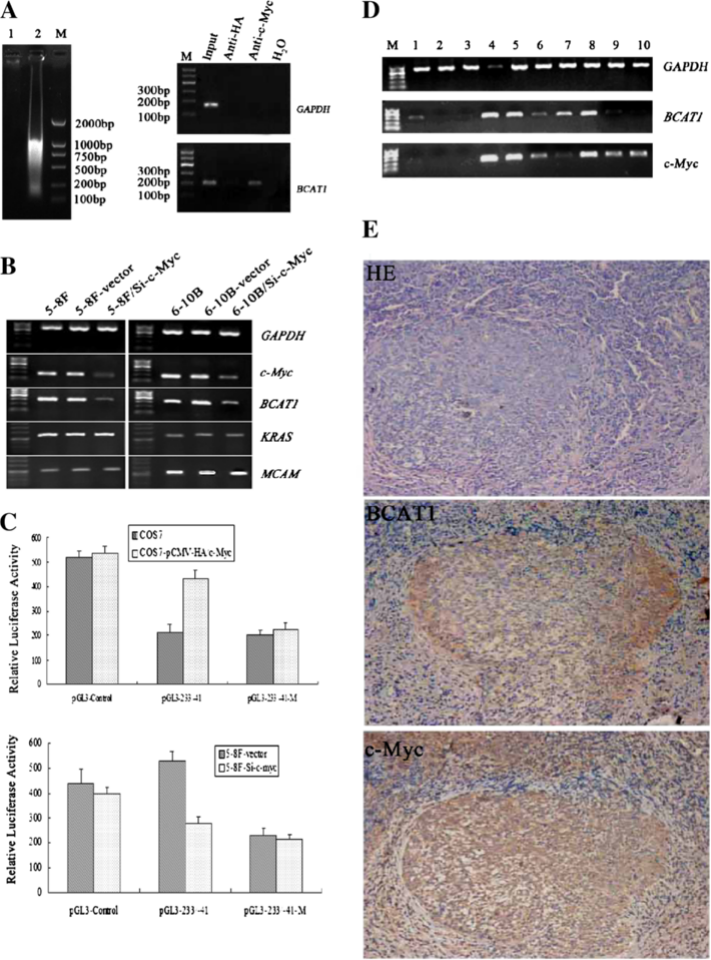
**The regulation of *BCAT1 *by c-Myc.** (**A**) ChIP confirmed that transcription factor c-Myc can specifically bind to the regulatory region of *BCAT1*. Lane 1 and 2 represent gDNA untreated or treated with ultrasonication, respectively. (**B**) Detection of the mRNA level of *BCAT1* in 5-8F cells and 6-10B cells transfected with pRNAT-U6.1/Si-c-Myc vector or blank vector. *BCAT1* mRNA level was reduced when the endogenous expression of *c*-*Myc* was blocked both in 5-8F cells and 6-10B cells, while the expression of *KRAS* or *MCAM*, two non-target genes of c-Myc, was stable despite the change of c-Myc’s level in these cells. (**C**) Luciferase reporter assay demonstrated the influence of c-Myc on *BCAT1* promoter activity. The results showed that the luciferase activity was positively correlated to the expression level of c-Myc. Here, we used 5-8F-vector cells instead of 5-8F cells as control. (**D**) The co-expression of *BCAT1* and *c*-*Myc* was detected in NPC tissues by RT-PCR. Lanes 1–3 represent *BCAT1* and *c*-*Myc* expression in CN tissues. Lanes 4–10 represent *BCAT1* and *c*-*Myc* expression in NPC tissues. *GAPDH* was used as an internal control. (**E**) IHC analysis of the same batch of NPC biopsies demonstrated that BCAT1 and c-Myc were co-expressed in most NPC tissues.

By searching NNPP and TESS, a c-Myc recognition site (CACGTG) was discovered in the 5’ regulatory region of *BCAT1* gene, suggesting that expression of *BCAT1* may be regulated by the transcription factor c-Myc. ChIP experiment using anti-c-Myc antibody was carried out to co-precipitate DNA sequences binding to c-Myc. The specific primers at −233 to -41 bp of *BCAT1* were designed. As shown in Figure 3A, a 193 bp fragment of *BCAT1* sequence was amplified, indicating that c-Myc transcription factor can directly bind to the specific promoter region of *BCAT1* gene.

Subsequently, we analyzed the regulation of *BCAT1* by c-Myc through knocking down *c*-*Myc* expression in NPC cells. When *c*-*Myc* shRNA vectors were transfected into 5-8F and 6-10B NPC cells, the mRNA expression of *c*-*Myc* decreased by 80% and 70% in 5-8F-Si-c-Myc and 6-10B-Si-c-Myc cells, respectively, as measured by semi-quantitative RT-PCR. As expected, the expression of *BCAT1* was also inhibited by 85% and 72% in 5-8F-Si-c-Myc and 6-10B-Si-c-Myc cells, respectively. Meanwhile, the expression of *KRAS* and *MCAM*, two c-Myc non-target genes, was unaffected by c-Myc knockdown (Figure 3B), further supporting that c-Myc can regulate *BCAT1* expression.

To further test whether c-Myc regulates *BCAT1* expression, we performed luciferase assay. The COS7 cells with absent expression of *c*-*Myc* were co-transfected with pGL3-233/-41 recombinant and c-Myc expression vector. The results showed that the luciferase activity of reporter system in co-transfected cells was markedly higher than that in parental COS7 cells and COS7 cells transfected with pGL3-233/-41-M mutant in which the c-Myc binding site was mutated (Figure 3C). We also conducted the luciferase assay in 5-8F-Si-c-Myc cells. Similarly, once c-Myc was knocked down in 5-8F cells, luciferase activity of pGL3-233/-41 recombinant dramatically decreased (Figure 3C), but that of pGL3-233/-41-M mutant had no significant change. Together, these results indicate that c-Myc directly binds to promoter of *BCAT1* and transactivates its expression.

### Expression of *c*-*Myc* and *BCAT1* was detected in NPC tissues

The mRNA expression of *c*-*Myc* and *BCAT1* was detected by RT-PCR in 6 chronic nasopharyngitis (CN) samples and 28 NPC samples. The results showed that *c*-*Myc* and *BCAT1* mRNA expression were low or undetectable in 6 CN tissues, while over expression of *c*-*Myc* and *BCAT1* was found in 67.9% (19/28) and 64.3% (18/28) of NPC tissues, respectively. In addition, *c*-*Myc* and *BCAT1* exhibited the same mRNA expression patterns in 74% of NPC tissues, as they were lowly expressed in 21% (6/28) and co-upregulated in 53% (15/28) of NPC tissues (Figure 3D, Table 2).

**Table 2:** Correlation analysis between c-Myc and BCAT1 expression in the same batch of NPC tissues

	**c**-**Myc**						
**BCAT1**		**RT-PCR**			**IHC**		
		L	U	Total	L	U	Total
	L	6	4	10	4	3	7
	U	3	15	18	2	13	15
	Total	9	19	28	6	16	22
		*P* = 0.019			*P* = 0.032		

*U* up-expression, *L* low expression.

The protein expression of c-Myc and BCAT1 was also examined by IHC in 22 NPC samples. The c-Myc or BCAT1 protein was positively stained in 73% (16/22) and 68% (15/22) of NPC tissues, respectively. Among them, c-Myc and BCAT1 were simultaneously and positively stained in 59% (13/22), whereas lowly or negatively in 18% (4/22) of NPC tissues (Figure 3E, Table 2).

The results showed a positive correlation of *c*-*Myc* expression and *BCAT1* expression in NPC tissues (Table 2, *P* = 0.019 for RT-PCR; *P* = 0.032 for IHC).

### Silencing *BCAT1* inhibited colony formation, migration and invasion of NPC cells

**Figure 4:**
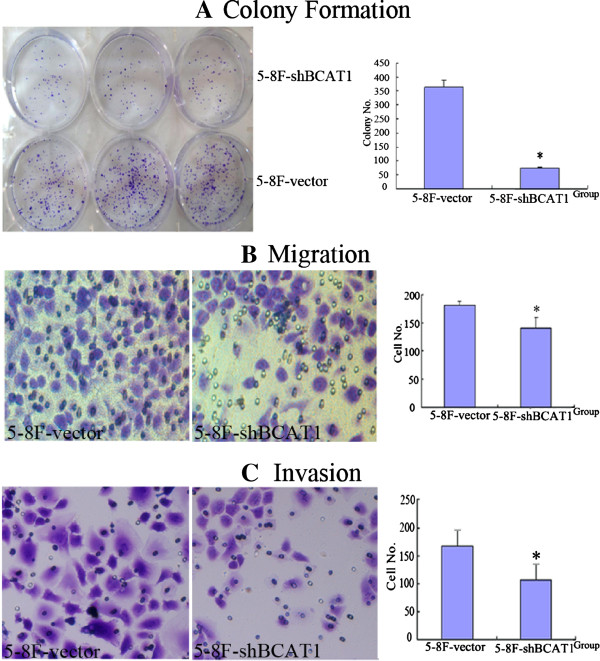
**Detection of the colony formation ability, migration and invasion capacities of NPC cells.** The colony formation ability (**A**), migration (**B**) and invasion capacities (**C**) of 5-8F cells decreased when the expression of *BCAT1* was blocked.

The cell growth of 5-8F NPC cells stably transfected with BCAT1-shRNA (5-8F-shBCAT1) and empty vector (5-8F-vector) was observed using clonogenesis assay. The colony formation ratios of 5-8F-shBCAT1 and 5-8F-vector cells were 10.7% ± 0.5% and 52.1% ± 3.5%, respectively (Figure 4A), demonstrating that *BCAT1* is critical for maintenance of NPC cell growth.

Using the migration assay, cell mobility was analyzed in 5-8F-shBCAT1 and 5-8F-vector cells. 5 × 10^4^ cells were inoculated on the filter membrane and cultivated for 18 hrs. Figure 4B showed that as measured by the numbers of cells migrating through the filter membrane, 5-8F-shBCAT1 cells (141.67 ± 17.9) demonstrated a noticeable decrease in the mobility compared to 5-8F-vector cells (180.8 ± 7.35).

The invasion capability associated with *BCAT1* expression was examined with matrigel-coated transwell chambers. The 5-8F-shBCAT1 and 5-8F-vector cells were inoculated on matrigel-coated membrane and cultivated for 48 hrs. The numbers of cells migrating through the membrane were 105 ± 33 and 168 ± 29.35, respectively (Figure 4C). Clearly, down-regulation of *BCAT1* remarkably impairs NPC cell invasion.

## Discussion

CGH-array is a newly developed technique for detecting genetic lesions in cancer and other diseases [18]. Numerous genetic abnormalities have been identified in multiple chromosomal regions in NPC tissues and cell lines [19]. Frequent gains on 1q, 3q, 8q, 11q, 12p and 12q, and losses on 3p, 9p, 11q, 14q and 16q, have been found. Moreover, several minimal regions of gains including 3q27.3-28, 8q21-24 and 11q13.1-13.3 have been identified and several minimal deleted regions have been mapped to 3p14.1-22, 11q13.3-24, 13q14.3-22, 14q24.3-32.1 and 16q22-23 [19]. We have analyzed 170 comparative genomic hybridization (CGH) samples and constructed a tree model to predict NPC tumorigenesis. We are particularly interested in the gain of 12p11-12 (+12p11-12) since +12p11-12 is a region frequently amplified and may be an early event in the development of NPC [15].

*BCAT1* is located at 12p12.1, and codes for the cytosolic form of branched-chain amino acid transaminase which catalyzes the reversible transamination of branched-chain alpha-keto acids to branched-chain L-amino acids essential for cell growth. *BCAT1* has been reported to be highly conserved in evolution and disruption of its yeast homolog affects cell growth [20,21]. Several groups have confirmed that *BCAT1* is involved in cell proliferation, cell cycle progression, differentiation and apoptosis, and plays an important role in several malignancies, especially in the progression of nonseminomas [22-24]. The mouse homologue of *BCAT1* has been shown to be amplified and overexpressed in a teratocarcinoma cell line [25]. Retroviral transduction of *BCAT1* into fetal rat brain cells with SV40 large T-antigen induced tumor formation with characteristic features of medulloblastoma [26].

Previously, RT-PCR results have presented that *BCAT1* is significantly up-regulated in NPC tissues and silencing its expression blocks NPC cell proliferation and the G1/S transition, indicating that high expression of *BCAT1* may play an important role in NPC cell survival [16]. Here, we further performed IHC analysis of different stages of NPC and found that BCAT1 protein level increased in the low-to-moderate grade atypical hyperplasia tissues as well as high-grade atypical hyperplasia tissues, *in situ* and invasive carcinomas, suggesting that *BCAT1* overexpression may be an important early event in NPC occurrence and maintain throughout NPC progression. There are several factors that can account for the abnormalities of gene expression, such as gene mutation, DNA amplification, transcriptional regulation and epigenetic changes, alone or synergistically. Gene mutation and amplification are two common causes for genetic activation of oncogenes. It is well known that *Ras* mutation is closely related to various malignancies such as breast cancer and lung cancer [27], and *TRK* mutation is also found to be associated with neuroblastoma [28]. *HER*-*2*/*neu* amplification is frequently detected in node-negative breast carcinoma tissues and it is a good example for oncogene activation by gene amplification [29]. We first analyzed whether *BCAT1* has mutation by sequencing 11 exons of *BCAT1* in 20 cases of NPC. Only one SNP site in exon1 was detected, suggesting that gene mutation of *BCAT1* is a rare incident in NPC. By using real-time PCR, we also analyzed three microsatellite loci including D12S1435, D12S1617 and RH44650 to examine whether *BCAT1* is amplified in NPC. Our results demonstrated that 42.4% (12/28) of NPC tissues manifested amplification, revealing that *BCAT1* overexpression may be due to its amplification in a portion of NPC tissues. Genes such as *CDH13*[30], *p16* and *p27*[8] have been reported to be involved in the early development of NPC. *BCAT1* over-expression, together with abnormal expression of *CDH13*, *p16*, *p27* and others, may result in transition from normal epithelia to hyperplastic epithelia.

*BCAT1* was first identified from a c-Myc-induced tumor and has been proven to be directly regulated by c-Myc through its binding to the specific DNA sequence, CACGTG [17,25]. C-Myc is an oncogene and transcription factor involved in the tumorigenesis of multiple cancers, such as Burkitt’s lymphoma and breast cancer [17]. Both *BCAT1* and *c*-*Myc* were found to be overexpressed in NPC [31]. We thus used IHC, RNAi, ChIP and Luciferase reporter system to investigate whether *BCAT1* is directly regulated by c-Myc in NPC. 59% of NPC tissues were double positive for c-Myc and BCAT1. Silencing the endogenous expression of *c*-*Myc* by RNAi also decreased *BCAT1* mRNA level in 5-8F-Si-c-Myc and 6-10B-Si-c-Myc cells. Using luciferase assay, we found transcription factor c-Myc up-regulated *BCAT1* expression. Furthermore, we confirmed that c-Myc can directly bind to the *BCAT1* promoter. Our results revealed that c-Myc, together with *BCAT1* amplification, up-regulates *BCAT1* expression and leads to *BCAT1* activation in NPC tissues.

One of the major clinical features of NPC is early metastasis. Several genes have been found to be associated with the metastasis of NPC, for example, *LMP1*, *LMP2A*, *p16*, *nm*-*23*, *CD44v6*, *TSLC1*, *NGX6*, *MMP9* and *LTF*[6,9,32-35]. However, they cannot fully elucidate the mechanisms underlying NPC metastasis. In this study, we indicated increased expression of *BCAT1* in the premalignant and NPC tissues. By performing colony formation, migration and invasion assays, we showed that colony formation, cell mobility and invasion abilities of 5-8F cells were reduced in response to knockdown of *BCAT1* expression. Consistent with our data, high *BCAT1* expression is associated with a high incidence of metastasis resulting in an adverse disease-free survival in colorectal adenocarcinomas [36]. Both mRNA and protein levels of *BCAT1* are higher in medulloblastoma patients with metastasis compared with those without metastasis (*P* < 0.01) [37]. Taken together, BCAT1 may be a favorable biomarker to indicate NPC early metastasis.

## Conclusion

In summary, for the first time, we demonstrate that expression of *BCAT1*, which locates in the frequently amplified 12p12 region, increases at early pathological stage of NPC. Gene amplification is an important cause for overexpression of *BCAT1* in NPC, while c-Myc also plays a critical role in regulation of *BCAT1* expression. We also confirm that high expression of *BCAT1* is associated with the mobility of NPC cells, indicating that it may be a promising target for NPC diagnosis and treatment.

## Methods

### Cell culture

5-8F, 6-10B and COS7 cells were cultured in RPMI1640 (Gibco BRL, Bethesda, MD) media with 10% fetal bovine serum (FBS) at 37°C in an atmosphere containing 5% CO_2_. The NPC cell lines 5-8F and 6-10B were derived from the same NPC cell line SUNE-1. Although sharing almost the same genetic background, the two NPC cell lines have different metastatic capability, for 5-8F cell line had high metastasis potential, while 6-10B cell line was non-metastatic [38].

### Patients and tissues

Six chronic nasopharyngitis (CN) biopsies and 28 primary poorly-differentiated NPC biopsies were obtained from CN and NPC patients with consent before treatment at Hunan Tumor Hospital (Changsha, Hunan, China), Xiangya Hospital of Central South University (CSU), the Second Xiangya Hospital of CSU and the Third Xiangya Hospital of CSU (Changsha, Hunan, China) in 2006 and 2007.

A total of 120 paraffin-embedded specimens, including 7 normal nasopharyngeal epithelia samples, 24 mild or moderate atypical hyperplasia samples, 9 severe atypical hyperplasia samples and 80 NPC samples, were supplied by Hunan Tumor Hospital and the Second Xiangya Hospital of CSU. All the specimens were stained with haematoxylin and eosin (HE) for histological examination and reviewed by an otorhinolaryngologic pathologist. The present study was approved ethically by Cancer Research Institute review board of CSU. All patients provided informed written consent.

### Immunohistochemistry (IHC) staining

Investigating the expression of *BCAT1* in different stages of precancerous lesions can help us evaluate the significance of this gene in NPC pathogenesis. Therefore, we used IHC method to analyze the expression of BCAT1 protein in the normal nasopharyngeal epithelia including pseudo-stratified ciliated epithelia and stratified epithelia, low-to-moderate grade atypical hyperplasia tissues, high-grade atypical hyperplasia tissues and NPC tissues, according to the protocol described in our previously published paper [39]. Meanwhile, the co-expression of BCAT1 and c-Myc in NPC tissues was also detected by IHC. Incubation with anti-BCAT1 (BD, Franklin Lakes, NJ) or anti-c-Myc (Calbiochem, Darmstadt, Germany) was carried out overnight at 4°C. Semi-quantitative assessment of BCAT1 and c-Myc immunostaining was performed by consensus and comprised both intensity of staining (0, 1, 2, or 3) and extent of staining (0, 0%; 1, <10%; 2, 10-50%; 3, >50%). The scores for the intensity of staining and extent of staining were multiplied to give a weighted BCAT1 or c-Myc score for each case (maximum possible, 9). The cases with at least moderate staining intensity (2 or 3) in a minimum of 10% of tumor cells were regarded as BCAT1 or c-Myc positive (++ or +++, total weighted score of > 4 out of 9), while the cases with weighted score of 0 (−) or 1–3 (+) were regarded as BCAT1 or c-Myc negative. BCAT1 immunostaining in normal or hyperplastic nasopharyngeal epithelia was similarly assessed. All of the biopsy samples were detected under the exactly same condition.

### Detection of exon mutation of *BCAT1* in NPC tissues

The primers for all the 11 exons of *BCAT1* were designed by Primer 5 software and synthesized by Invitrogen (Shanghai, China). PCR was carried out using the genomic DNA from NPC tissues and the matched blood samples as templates. Then the PCR products were sequenced after being purified. The primer sequences are listed in Table 3.

**Table 3:** **Primers for amplifying 11 exons of *BCAT1***

**Primer**	**Sequence (5’-3’)**	**Product size (bp)**
*Exon 1*	F-GGGGAGCAGCCTTAGTGT	456
	R-GAGTGGAGGTTAAACCGAAA	
*Exon 2*	F-TACCCACCTGCATTTACTT	583
	R-TCAACGTGCTTTGTTTCTC	
*Exon 3*	F-TAATCTAGCCAGCGAATG	311
	R-GTACCCACAGTGAAGTGC	
*Exon 4*	F-GATGAACGCCCATAGGAA	251
	R-CCGTGACCCGTTACATTA	
*Exon 5*	F-ATTGCCACATTGTGAGAAA	417
	R-GTATGGTAAGAGGTAGGGA	
*Exon 6*	F-AAGTATGGTAATAGCTCCTG	352
	R-ATGGCACTAACTAAATGGTC	
*Exon 7*	F-GGGGATGAAGTATGTTTG	250
	R-GTCTTTCTGGTCCTGTTG	
*Exon 8*	F-ATGCCTAATGTAGTGAAAG	478
	R-ACAGACTTGGGAAGTTAA	
*Exon 9*	F-GCCACTTCCAGCTTTCCC	385
	R-GCATCTTGGGTCTGGGTC	
*Exon 10*	F-CTTCAGTGGAATTGCCTTAG	375
	R-TTTCCCATTTCTGCTTTG	
*Exon 11*	F-TCAAAGCAGAAGCGAACC	251
	R-GTAGCCAAAGAAATCTATCACA	

### Real-time quantitative PCR (qPCR) and reverse transcription PCR (RT-PCR)

The qPCR was performed on a Bio-Rad iQ5 system (Hercules, CA) with SYBR Green I PCR kit (TaKaRa, Dalian, China) to quantitatively analyze *BCAT1* amplification in NPC tissues. Three microsatellite loci located in the *BCAT1* gene were selected for this analysis. Gene amplification was set as 2^-ΔΔC^ >2.0 compared with control. Semi-quantitative RT-PCR was performed to detect the mRNA expression levels of *BCAT1* and *c*-*Myc* in CN tissues, NPC tissues and cell lines. Total RNA was extracted with TRIzol reagent (Invitrogen, Carlsbad, CA). cDNA was synthesized from DNase I-digested RNA (2 μg) using oligo(dT) as the primer with a commercially available reverse transcription system (Promega, Madison, WI) according to the manufacturer’s protocol. Glyceraldehyde-3-phosphate dehydrogenase (*GAPDH*) was amplified as an internal control. Due to small size of NPC biopsies, the NPC samples for RT-PCR and IHC were not the same batch. The primer sequences for qPCR or RT-PCR are listed in Table 4.

**Table 4:** Summary for primer sequences and product sizes

** Primer**	** Sequence (5’-3’)**	**Product size (bp)**
Primers for RT-PCR		
*GAPDH*	F:5'-CCACCCATGGCAAATTCCATGGCA-3'	550
	R:5'-TCTAGACGGCAGGTCAGGTCCACC-3'	
*BCAT1*	F: 5'-CCAAAGCCCTGCTCTTTGTA-3'	305
	R: 5'-TGGAGGAGTTGCCAGTTCTT-3'	
*c*-*Myc*	F: 5'-CCTACCCTCTCAACGACAGC-3'	179
	R: 5'-TTCCTCCTCAGAGTCGCTGC-3'	
*KRAS*	F: 5'-GCAAAGACAAGACAGGGTG-3'	264
	R: 5'-GGTAAAAGCTAACAGTCTGC-3'	
*MCAM*	F: 5'- CTCCGCGTCTACAAAGCTCC-3'	213
	R: 5'- ACCACTCGACTCCACAGTCT-3'	
Primers for microsatellite loci of *BCAT1*		
D12S1435	F-CTTGTGCAACCCTCCCAC	198
	R-ATATGTGCTGTGAATACATCCACC	
D12S1617	F-AGCCTGAGGGGCCACAT	259
	R-TGGGCAACTTGGATAAGAAACA	
RH44650	F-AAGAATGTGTCTATTGCCAGCA	146
	R-CTCATGCCTCTGAAGGTTTTG	

### Chromatin immunoprecipitation (ChIP) assay

ChIP assay was performed with EZ-ChIP™ kit (Millipore, Darmstadt, Germany). Two specific primers (5’-TGGCATAGCACTGAAAGG-3’ and 5’-CTGACTGGCAGTTGGTTG-3’) were used to amplify a 193 bp fragment containing the predicted c-Myc binding site in the *BCAT1* regulatory region. As a negative control, *GAPDH* was also amplified with the corresponding primers (5’-CGACCACTTTGTCAAGCTCA-3’ and 5’-AGGGGTCTACATGGCAACTG-3’).

### Plasmids and recombinants

The plasmids including pGL3-control, pGL3-promoter and pRL-TK used for luciferase reporter gene expression analysis were purchased from Promega Ltd. Vector for knocking down c-Myc expression (pRNAT-U6.1/Si-c-Myc) and c-Myc expression vector (pCMV-HA/c-Myc) were both presented by Dr. Huaying Liu from our institute. For cloning pRNAT-U6.1/Si-c-Myc, a vector expressing shRNA, an oligonucleotide encoding a stem-loop structure targeting *c*-*Myc* with the targeting sequence AGACTCTGACACTGTCCA, was designed and then subcloned into the pRNAT-U6.1 vector (Genscript, Piscataway, NJ) under the control of the U6 promoter. pGL3-233/-41 vector was constructed by amplifying a 193 bp fragment comprising −233 ~ −41 bp upstream of *BCAT1* transcription start site (TSS) which contained the predicted c-Myc binding site (CACGTG) and inserting it into pGL3-promoter vector. Meanwhile, pGL3-233/-41-M vector with a mutated c-Myc binding site (CGCGTT) in the *BCAT1* regulatory region was also constructed. Key regions in all constructs were verified by DNA sequencing.

### Knockdown of *c*-*Myc* in NPC cell lines

5-8F-Si-c-Myc and 6-10B-Si-c-Myc cell lines with suppressed endogenous *c*-*Myc* expression were established by introducing pRNAT-U6.1/Si-c-Myc vector into 5-8F cells and 6-10B cells, respectively. For comparison, 5-8F-vector and 6-10B-vector cell lines were also yielded by transfecting pRNAT-U6.1 blank vector into 5-8F cells and 6-10B cells. Stable transfection was performed using Lipofectamine™ 2000 reagent (Invitrogen) following the manufacturer’s instructions. G418 (500 μg/ml, Millipore) was used to select the stable clones. The mRNA expression levels of *c*-*Myc*, *BCAT1*, *KRAS* and *MCAM* in NPC cell lines were detected by RT-PCR as previously described. Since the optimal PCR amplification parameters of them were not identical, we examined their expressions in different tubes. *GAPDH* was used as endogenous reference gene for normalizing variance in the quality of RNA and the amount of input cDNA. The same volume of PCR products was used to be analyzed by electrophoresis on the same agarose gel. The intensity of each band was measured by Image Master VDS (Pharmacia Biotech, Piscataway, NJ) and was analyzed by Bandleader software version 3.0. The expression levels of *c*-*Myc*, *BCAT1*, *KRAS* and *MCAM* in NPC cells were evaluated after they were normalized by transforming them into the ratio of the band intensity of each gene over that of *GAPDH* of the same samples. Each sample was repeated in triplicate. The primer sequences for RT-PCR are listed in Table 4.

### Luciferase activity assay

COS7 cells with no endogenous *c*-*Myc* expression, 5-8F-Si-c-Myc cells with inhibited *c*-*Myc* expression, 5-8F-vector cells with endogenous *c*-*Myc* expression, were plated in 24-well plates at a density of 1 × 10^4^ cells/well. After 24 hrs, pGL3-233/-41 (or pGL3-233/-41-M), pCMV-HA/c-Myc and pRL-TK vectors were introduced into COS7 cells at a ratio of 10:10:1, and pGL3-233/-41 (or pGL3-233/-41-M) and pRL-TK vectors were co-transfected into 5-8F-Si-c-Myc and 5-8F-vector cells at a ratio of 10:1 by Fugene 6 transfection reagent (Roche, Switzerland). Another 36 hrs later, cells were washed twice, suspended in 500 μl reporter lysis buffer (Promega), and then the firefly luciferase activity was measured using the dual luciferase reporter assay system and a GloMax 20/20 luminometer (Promega) according to the manufacturer’s protocol. The Renilla luciferase vector pRL-TK (Promega) was co-transfected to standardize transfection efficiency in each experiment. As a positive control, the pGL3-control vector was also co-transfected into COS7 cells with pCMV-HA/c-Myc and pRL-TK vectors at a ratio of 10:10:1 or co-transfected into 5-8-Si-c-Myc and 5-8F-vector cells with pRL-TK vector at a ratio of 10:1.

### Colony formation assay

Colony formation assay was conducted as described in our published paper [12] with minor modification. 5-8F, 5-8F-shBCAT1 and 5-8F-vector cells were seeded in six-well plates at the density of 700 cells per well. 5-8F-shBCAT1 and 5-8F-vector cells were established in our previous work [16]. After incubation for 8 days at 37°C in a 5% CO_2_ incubator, the cells were fixed with methanol and stained with crystal violet. Colonies containing at least 50 cells were counted under inverse microscope (Nikon, Japan). Colony formation ratio was also calculated.

### Cell migration and invasion assays

The *in vitro* migration and invasion abilities were compared between 5-8F-shBCAT1 and 5-8F-vector cells by using transwell chambers and matrigel-coated invasion chambers (Corning, Tewksbury, MA). For invasion assay, 8 μm pore transwell inserts coated with matrigel in cold serum-free media were seeded with 5 × 10^4^ cells per well and incubated for 48 hrs. Non-invasive cells on the upper surface of the filter were removed by wiping with a cotton swab, and cells that migrated through the membrane and stuck to the lower surface of the membrane were fixed with 10% paraformaldehyde and stained with 0.1% hexamethylpararosaniline for 30 mins. For quantification, the cells were counted in five predetermined fields under a microscope. Data were expressed as the average number of cells migrating through the filters. The procedures of migration assay were similar to those described in matrigel invasion assay except there was no matrigel, the incubation time was 18 hrs and the fixative was methanol.

### Bioinformatic analysis

Some bioinformatics tools such as Neural Network Promoter Prediction (NNPP) (http://www.fruitfly.org/seq_tools/promoter.html) and Transcription Element Search Software (TESS) (http://www.cbil.upenn.edu/tess) were used to predict the possible regulatory relationship and interaction mode between c-Myc and *BCAT1*. SNPs database (http://www.ncbi.nlm.nih.gov/snp/) was utilized to discriminate between mutation and SNP of *BCAT1*.

### Statistical analysis

Statistical analysis was performed using Wilcoxon rank sum test, chi-square test, and student *t*-test. In all analyses, SPSS 13.0 statistical software was used and the statistical significance level was set at *P* < 0.05.

## Abbreviations

CGH: Comparative genomic hybridization; ChIP: Chromatin immunoprecipitation; CN: Chronic nasopharyngitis; CSU: Central South University; HE: Haematoxylin and eosin; IHC: Immunohistochemistry; NNPP: Neural network promoter prediction; NPC: Nasopharyngeal carcinoma; qPCR: Real-time quantitative PCR; RNAi: RNA interference; RT-PCR: Reverse transcription PCR; SNP: Single nucleotide polymorphism; TESS: transcription element search software; TSG: Tumor suppressor gene; TSS: Transcription start site.

## Competing interests

The authors declare that they have no competing interests.

## Authors’ contributions

RC designed the general study, wrote the protocols, revised the manuscript and provided the funding. ZW and FX performed most of the experiments. JX, HW, Liu Z, Li Z, ZL and WL contributed to administrative, technical or material support (such as clinical samples collection). ZB, SJ, LJ and LY were in charge of the literature searches, analyses and partial experiments. WL undertook the statistical analysis. ZW and HW drafted the manuscript. YK provided critiques of the manuscript. All authors read and approved the final manuscript.
